# Synergistic Promotion of Photocatalytic Degradation of Methyl Orange by Fluorine- and Silicon-Doped TiO_2_/AC Composite Material

**DOI:** 10.3390/molecules28135170

**Published:** 2023-07-02

**Authors:** Jinyuan Zhu, Yingying Zhu, Yifan Zhou, Chen Wu, Zhen Chen, Geng Chen

**Affiliations:** 1 Faculty of Maritime and Transportation, Ningbo University, Ningbo 315211, China; 2111084046@nbu.edu.cn (J.Z.); 2111087081@nbu.edu.cn (Y.Z.); 2011084024@nbu.edu.cn (Z.C.); chengeng@nbu.edu.cn (G.C.); 2 Ningbo Energy Group Co., Ltd., Ningbo 315000, China; wuchen_nbzj@163.com

**Keywords:** F-Si-TiO_2_, methyl orange, visible light, doped, electronic cage

## Abstract

The direct or indirect discharge of organic pollutants causes serious environmental problems and endangers human health. The high electron–hole recombination rate greatly limits the catalytic efficiency of traditional TiO_2_-based catalysts. Therefore, starting from low-cost activated carbon (AC), a photocatalyst (F-Si-TiO_2_/AC) comprising fluorine (F)- and silicon (Si)-doped TiO_2_ loaded on AC has been developed. F-Si-TiO_2_/AC has a porous structure. TiO_2_ nanoparticles were uniformly fixed on the surface or pores of AC, producing many catalytic sites. The band gap of F-Si-TiO_2_/AC is only 2.7 eV. In addition, F-Si-TiO_2_/AC exhibits an excellent adsorption capacity toward methyl orange (MO) (57%) in the dark after 60 min. Under the optimal preparation conditions, F-Si-TiO_2_/AC showed a significant photodegradation performance toward MO, reaching 97.7% after irradiation with visible light for 70 min. Even under the action of different anions and cations, its degradation efficiency is the lowest, at 64.0%, which has good prospects for practical application. At the same time, F-Si-TiO_2_/AC has long-term, stable, practical application potential and can be easily recovered from the solution. Therefore, this work provides new insights for the fabrication of low-cost, porous, activated, carbon-based photocatalysts, which can be used as high-performance photocatalysts for the degradation of organic pollutants.

## 1. Introduction

In the process of pursuing rapid economic development, human beings have neglected to protect the environment, resulting in serious damage to the ecological environment, which poses serious threat to human health. The production processes used in the textile, food, and leather industries are complex, and many raw materials, especially azo dyes, are directly or indirectly lost to water bodies, causing pollution. The presence of azo dyes will affect light projection, reduce the photosynthesis of aquatic plants, and further affect the survival of aquatic organisms [1,2]. For humans, drinking contaminated water or their accumulation through the food chain can induce cancer or lead to organ failure [3,4,5]. Therefore, there is an urgent need to develop an efficient, energy-saving, non-secondary-pollution-free, and effective method for removing azo dyes.

At present, the methods used to treat water pollution mainly include adsorption [6,7], precipitation [8], biological treatment [9,10], membrane separation [11,12], and advanced oxidation processes [13,14]. Physical methods such as adsorption, precipitation, and membrane separation usually cannot be recycled and are prone to secondary pollution in the process of processing materials [15]. Biological treatment methods have a limited range of use due to their harsh requirements for environmental temperature, pH, etc. [16]. As one of the AOPs reported to date, photocatalysis is considered to be an ideal method because of its low cost, environmental protection, high efficiency, and mineralization and degradation of pollutants [17,18]. Titanium dioxide (TiO_2_), as the most common photocatalytic material reported to date, is widely used in the study of azo dyes. However, pure TiO_2_ has obvious limitations in photocatalysis [19,20,21]: (1) Its wide band gap of 3.2 eV leads to limited visible light absorption capacity. (2) Nano-TiO_2_ particles are difficult to recover and difficult to reuse. (3) The adsorption capacity is limited, and it is difficult to become fully in contact with pollutants. (4) The photogenerated electron–hole pairs recombine rapidly, the carriers cannot reach the surface of the catalyst, and fewer active radicals are generated.

Therefore, many efforts have been made in recent years to increase their photocatalytic potential. Among these methods, the introduction of non-metals, such as nitrogen [22], carbon [23], sulfur [24], and F [25], into TiO_2_ has proved to be one of the most feasible approaches reported to date. It has been reported that doping F anions into the TiO_2_ lattice can lead to an increase in the number of acidic sites, providing additional surface chemisorption centers for the reactants and facilitate the adsorption of organic molecules [26,27]. Moreover, the introduction of F ions into TiO_2_ results in the formation of Ti^3+^ that serves as the active site for the surface reactions. This can enhance the photocatalytic performance of F-doped TiO_2_ significantly [28,29]. According to the calculations and experimental findings of Ta et al. [30], the Si atoms may function as electron transport bridges between the materials, facilitating carrier separation while impeding electron–hole recombination. In a study conducted by Long et al. [31], co-doping of N and Si was shown to induce longer wavelengths of visible light to those induced by N or Si single doping. In general, these findings suggest that the co-doping F and Si represents an effective approach to expand the optical absorption edge of TiO_2_ into the visible light region, thereby enhancing its photocatalytic activity.

In this paper, a photocatalyst comprising F and Si ion co-doped TiO_2_ was loaded onto activated carbon (AC) (F-Si-TiO_2_/AC) via a one-step solvothermal method, which has the advantages of easy separation, strong adsorption capacity, high carrier transport capacity, and high photocatalytic activity. Methyl orange (MO) was used as a model azo dye pollutant to study the degradation of MO by F-Si-TiO_2_/AC. In order to study whether F-Si-TiO_2_/AC has potential value in actual wastewater treatment, the photocatalytic performance under different anions and cations was also carried out. F-Si-TiO_2_/AC has shown a very interesting phenomenon for the degradation of MO. Therefore, this study can provide reference for the design of new multi-ion co-doped photocatalysts.

## 2. Results and Discussion

### 2.1. SEM Analysis

**Figure 1:**
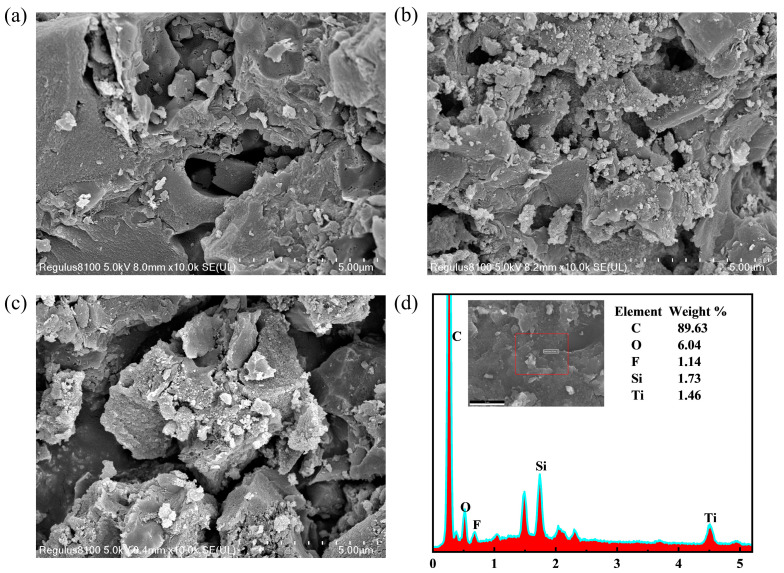
SEM images of (**a**) AC, (**b**) TiO_2_/AC, (**c**) F-Si-TiO_2_/AC, and (**d**) EDS images of F-Si-TiO_2_/AC.

The microstructures and morphologies of the synthetic samples (AC, TiO_2_/AC, and F-Si-TiO_2_/AC) were studied using SEM. Figure 1 shows that the surface of pure AC was irregular and exhibited developed porosity. As for the TiO_2_/AC samples, nano-TiO_2_ particles were not uniformly attached onto the AC surface with some agglomerations observed. Obviously, loading TiO_2_ onto AC helped to reduce the agglomeration of TiO_2_, thereby promoting the photocatalytic activity. In addition, a large number of nanoscale pores and cracks on the surface of AC were occupied by TiO_2_ and F-Si-TiO_2_, resulting in a reduction in the specific surface area of the composite [32]. Such observation is consistent with the nitrogen adsorption and pore size distribution characterization results. To further analyze the elemental composition, the relevant regions of F-Si-TiO_2_/AC were studied by EDS mapping. As shown in Figure 1d, the relative abundances of C, O, Si, Ti, and F were about 89.63, 6.04, 1.73, 1.46, and 1.14 wt%, respectively. These findings confirm the successful doping of various elements into the material.

### 2.2. XRD Analysis

**Figure 2:**
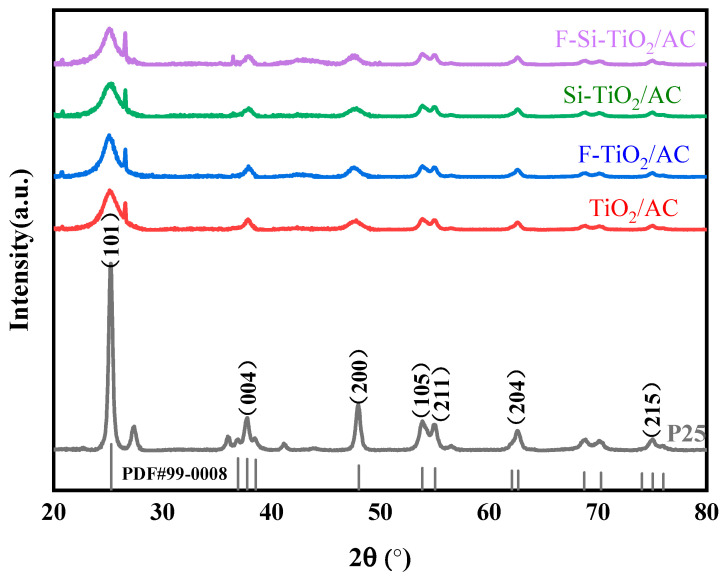
XRD patterns obtained for P25, TiO_2_/AC, F-TiO_2_/AC, Si-TiO_2_/AC, and F-Si-TiO_2_/AC.

The crystallographic structures of the catalysts were examined using XRD. Figure 2 shows XRD patterns obtained for the five samples (P25, TiO_2_/AC, F-TiO_2_/AC, Si-TiO_2_/AC, and F-Si-TiO_2_/AC). All of the samples exhibit similar XRD patterns with peaks at 2θ values of 25.3, 37.8, 48.0, 53.9, 55.1, 62.7, and 75.0°, corresponding to the (101), (004), (200), (105), (211), (204), and (215) planes of anatase TiO_2_ (PDF #99-0008), respectively [32]. Compared with bare TiO_2_/AC, no other new phases were observed in F-TiO_2_/AC, Si-TiO_2_/AC, and F-Si-TiO_2_/AC, indicating that the F and Si ions were physically adsorbed onto the surface of TiO_2_ (≡Ti-F and Si-O-Ti) and thus did not change the crystal phase and structure [33]. The crystallite sizes of the different samples based on the (101) peak of the XRD patterns were as follows: TiO_2_/AC (10.2 nm) > F-Si-TiO_2_/AC (9.4 nm) = F-TiO_2_/AC (9.4 nm) = Si-TiO_2_/AC (8.2 nm) [34,35]. The lower proportion of predominant facet and narrower widths of the XRD peaks in TiO_2_/AC revealed the good crystallinity (Table 1) [36,37]. Doping F and Si ions will increase the interplanar distance values of the material, which may be caused by F and Si ions doping into the unit cells of the material, resulting in unit cell deformation. The interplanar distance values are in accordance with the values obtained from earlier studies [38]. This result shows that F and Si ion doping can inhibit the growth of the TiO_2_ grains.

### 2.3. Nitrogen Physical Adsorption

**Figure 3:**
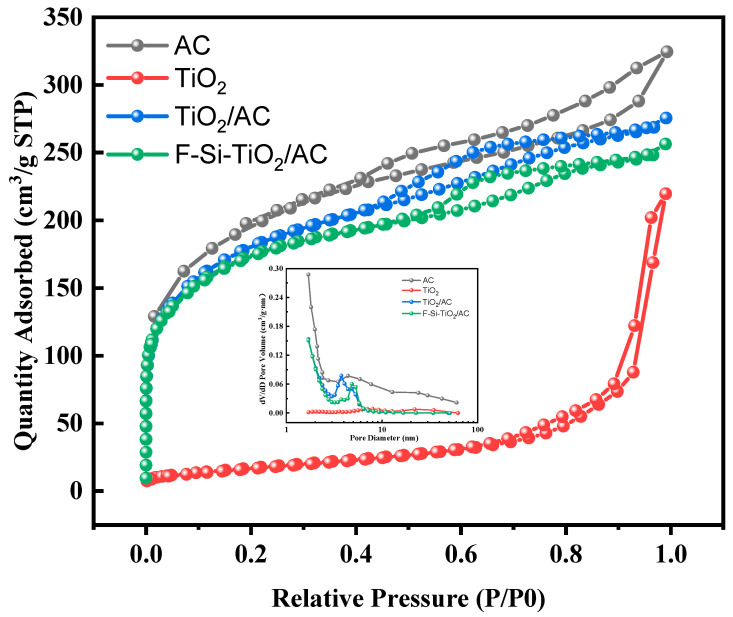
N_2_ adsorption–desorption isotherms and pore size distributions of AC, P25, TiO_2_/AC, and F-Si-TiO_2_/AC.

Furthermore, the surface area and pore diameter distribution are also important influencing factors on the photocatalytic performance. Figure 3 shows the nitrogen adsorption–desorption isotherms and pore size distribution curves obtained for the samples (AC, P25, TiO_2_/AC, and F-Si-TiO_2_/AC). The corresponding surface area (S_BET_), pore volume, and average pore size are listed in Table 2. AC, TiO_2_/AC, and F-Si-TiO_2_/AC exhibited typical type IV isotherms with H2-type hysteresis loops in the relative pressure range (P/P_0_) of 0.5–1.0, indicating that they are microporous and small mesoporous materials with complex pore sizes and distributions [39]. This is further confirmed by their narrow pore size distribution in the range of 1–10 nm. The specific surface area and average pore volume of AC are the largest, which are reduced after the addition of TiO_2_ due to the partial occupation of the pores of AC by TiO_2_. This is consistent with our SEM characterization results. TiO_2_/AC has a larger surface area when compared with F-Si-TiO_2_/AC, possibly due to the smaller grain size of TiO_2_ after F and Si doping, resulting in a decrease in specific surface area.

### 2.4. FTIR Spectroscopy

**Figure 4:**
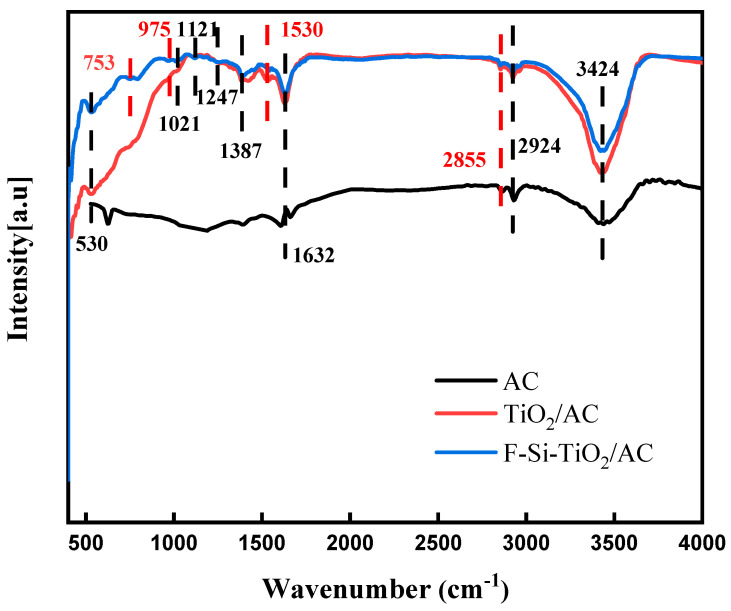
The FTIR spectra obtained for the TiO_2_/AC and F-Si-TiO_2_/AC samples.

The FTIR spectra obtained for the TiO_2_/AC and F-Si-TiO_2_/AC samples are shown in Figure 4. For TiO_2_/AC, the band at 3424 cm^–1^ can be attributed to the O-H, COOH on the surface of AC, and O-H telescopic vibrations of chemisorbed water. The peaks at 2924 and 2855 cm^–1^ belong to the symmetric and antisymmetric telescopic vibrations of the C-H bonds in the saturated -CH-, -CH_2_-, -CH_3_- alkyl groups, which are present in both samples. However, the peak intensity of the doped modified sample is smaller, indicating a minor reduction in the hydroxyl and carboxyl groups in the activated carbon after modification [40]. The absorption band at 1632 cm^–1^ can be attributed to the characteristic telescopic vibration peak of C=C in the carboxylic acid and lactone groups, and the absorption peak at 1530 cm^–1^ can be attributed to the contraction vibration absorption of the C=C bond in the activated carbon skeleton. The peaks at 530 and 1021 cm^–1^ correspond to the Ti-O and Ti-O-C bonds, respectively. For the F-Si-TiO_2_/AC sample, new peaks appear in the spectrum at 1121, 975, and 753 cm^−1^ upon the introduction of F and Si ions, which correspond to Si-O-Si, Si-O-Ti, and Ti-F, respectively [41]. This proves that the F and Si ions have been successfully doped into the material.

### 2.5. UV–vis Absorption Spectra and Photoluminescence Analysis

**Figure 5:**
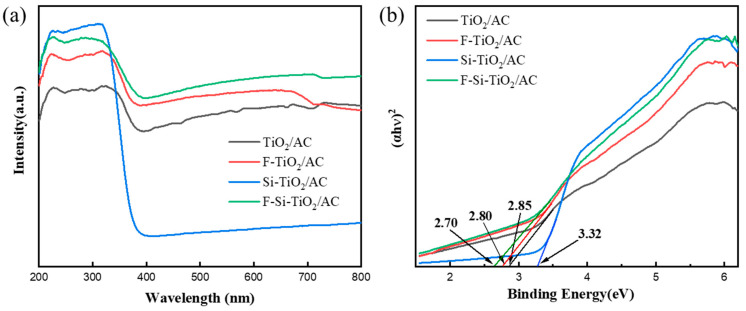
(**a**) UV–vis absorption spectra and (**b**) corresponding band gap energy (Eg) of TiO_2_/AC, F-TiO_2_/AC, Si-TiO_2_/AC, and F-Si-TiO_2_/AC.

It is well known that the optical absorption properties of a semiconductor play an essential role in their photocatalytic performance. Figure 5 shows the UV–vis absorption spectra and corresponding Kubelka–Munk plots obtained for TiO_2_/AC, F-TiO_2_/AC, Si-TiO_2_/AC, and F-Si-TiO_2_/AC. Figure 5a shows that the Si-doped sample exhibits a significant enhancement in its light absorption in the UV light region (200–400 nm) due to its band gap (∼3.32 eV) but a sharp decrease in the visible light region. As for F-TiO_2_/AC, the band gap energy is further decreased, which is attributed to oxygen vacancies and Ti^3+^. It has been reported that the F doping can convert some of the Ti^4+^ to Ti^3+^. The Ti^3+^ surface states in TiO_2_ form a donor level between the bandgaps of TiO_2_, which would enhance the visible light absorption [42,43]. The light absorption intensity of F-Si-TiO_2_/AC was greater than that of F-TiO_2_/AC at all wavelength ranges. This indicates the synergistic effect between the F and Si ions, which helps to improve the light absorption ability of the material. The band gap energy (Eg) of the prepared catalysts can be calculated using the Kubelka–Munk method [44]. Figure 5b shows that F-Si-TiO_2_/AC can effectively reduce the band gap energy of TiO_2_/AC from 2.85 to 2.70 eV, which facilitates the excitation of electrons from the valence band to the conduction band in F-Si-TiO_2_/AC under visible light irradiation, significantly enhancing the photocatalytic activity.

### 2.6. Photocatalytic Studies

**Figure 6:**
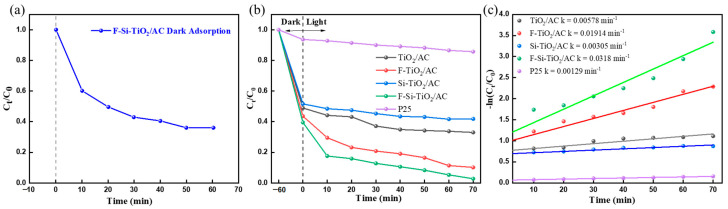
(**a**) Dark adsorption capability of F-Si-TiO_2_/AC, (**b**) photocatalytic degradation, and (**c**) degradation kinetic curves of MO under visible light irradiation and kinetic linear fitting of the degradation of MO under visible light irradiation.

It is necessary to apply a dark adsorption experiment on AC to determine when the adsorption–desorption equilibrium was established. The dark adsorption experiments on F-Si-TiO_2_/AC were performed for 60 min, and the results are shown in Figure 6a. After 50 min of stirring, the absorbance of the solution did not change any further, which indicates that the sample and contaminant had reached an adsorption equilibrium. Therefore, prior to irradiation with visible light, a 60 min dark adsorption period was applied to all photodegradation processes. The removal efficiency of the material in the dark adsorption process reaches 57%, attributed to the abundant presence of pores, hydroxyl groups, and carbon content on the surface of the AC. These properties enable the material to easily undergo physical or chemical adsorption of pollutants, making the material highly suitable for use in photocatalytic reactions.

#### 2.6.1. The Influence of Different Doping Elements

Figure 6b,c show the degradation and kinetic curves obtained for MO using the photocatalysts prepared with different doped elements. Figure 6b shows that only a small amount of MO was adsorbed on the surface of P25. However, after TiO_2_ was supported on AC, the adsorption capacity of the catalyst toward pollutants was significantly improved. This can be attributed to the strong adsorption capacity of AC. The degradation yields of MO on P25 under the visible light irradiation for 1 h are low (14.3%). In contrast to P25, Si-TiO_2_/AC showed a remarkably high photocatalytic activity of 58.17% for the degradation of MO. However, Figure 6c shows that the degradation rate of Si-TiO_2_/AC was between P25 and TiO_2_/AC. Since the absorption capacity of Si-TiO_2_/AC in the visible light region is weaker than TiO_2_/AC, we speculated that loading materials on the surface of AC will, on the one hand, reduce the agglomeration of materials and increase the contact area available to pollutants. On the other hand, it can increase the carrier transport capacity and reduce the recombination of electron holes. The photocatalytic degradation of MO by these composites follows the pseudo-first-order reaction kinetic model.

Although the absorption capacity of F-TiO_2_/AC was only slightly higher than that of TiO_2_/AC in the visible light region, F-TiO_2_/AC exhibits 1.6 times the degradation kinetic rate of TiO_2_/AC. This is because F doping increases the surface acidity of the material, which in turn enhances the adsorption of organic molecules on the F-TiO_2_/AC surface. Moreover, F doping has been reported to enhance the generation of free hydroxyl radicals (·OH) in F-TiO_2_/AC (Equation (1)) [45], which are formed via the oxidation of OH^–^ adsorbed on the TiO_2_ surface (OH_ads_) (Equation (2)), inducing a much lower reactivity than ·OH.

= Ti-F + H_2_O (or OH^−^) + h^+^_VB_ → =Ti-F + ·OH + H^+^ (1)

= Ti-OH^−^ (or Ti-OH_2_) + h^+^_VB_ → =Ti-OH_ads_ (or + H^+^) (2)

Interestingly, F-Si-TiO_2_/AC exhibited the highest photocatalytic activity with a degradation yield of 97.7% for the degradation of MO. The result may be attributed to synergetic effect of F and Si. Si reduces the compounding rate of photogenerated electron–hole pairs [46], while F enhances the production of OH_free_ via Equation (2). The rate constant for the photocatalytic degradation using the different samples was calculated using the Equation (4) and are summarized in Table 3.

#### 2.6.2. Effect of the Amount of F and Si Ion Doping

**Figure 7:**
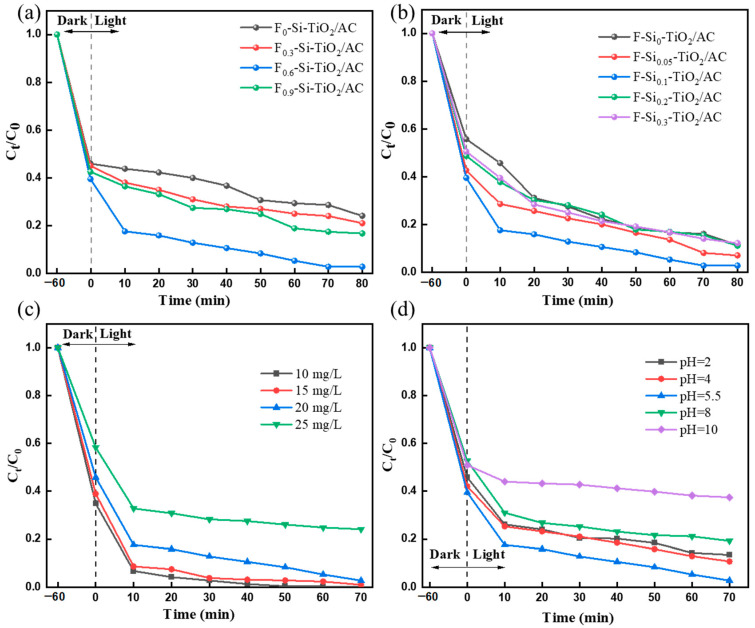
Photocatalytic efficiency toward MO after 70 min in the presence of (**a**) F_x_-Si-TiO_2_/AC and (**b**) F-Si_y_-TiO_2_/AC. (**c**) Photocatalytic efficiency observed for F-Si-TiO_2_/AC toward different concentrations of MO after 70 min. (**d**) Photocatalytic efficiency observed for F-Si-TiO_2_/AC toward MO under different pH conditions after 70 min.

The amount of elemental doping has the most significant effect on the properties of the photocatalyst. The effects of the amount of F and Si doping on the MO degradation efficiency were investigated and the results shown in Figure 7a,b. The MO degradation efficiency rapidly improved from 76.24% to 97.14% as the mass ratio of F:TiO_2_ increased from 0.0 to 0.6. However, the degradation efficiency suddenly dropped to 83% for MO when the mass ratio of F:TiO_2_ was 0.9. As the amount of F doping increases, more photoinduced electrons and holes are generated, which can produce more reactive oxygen species (·O^2−^and ·OH) to degrade more MO. However, F-TiO_2_ not only absorbs light but also reflects and scatters it. The light transmittance in the solution is decreased significantly when the mass ratio of F:TiO_2_ reached 0.9, resulting in a slight increase in the degradation efficiency. A similar phenomenon occurred upon Si doping. The MO degradation efficiency rapidly improved from 89.00% to 97.14% as the mass ratio of Si:TiO_2_ increased from 0 to 0.1. The MO degradation efficiency dropped to 89% and 88% when the mass Si:TiO_2_ ratio was 0.2 and 0.3, respectively. Therefore, the optimal mass ratio of F:Si:TiO_2_ was 0.6:0.1:1 in this study.

#### 2.6.3. The Effect of the Initial Concentration of MO Solution

The initial concentration of contaminants affects the light transmission capacity and thus the photocatalytic performance of the material. The effect of the initial MO concentration on the photocatalytic degradation efficiency was investigated using F-Si-TiO_2_/AC in solution. After 50 min of irradiation, the degradation efficiency of MO reached 99.6% at a low concentration (10 mg/L) (Figure 7c). However, the degradation efficiency decreased to 73.9% for MO after 50 min when the concentration was 25 mg/L. Obviously, the catalytic sites in F-Si-TiO_2_/AC could be covered with MO at high initial concentrations, leading to the deceleration of the generation of electron–hole pairs and the inhibition of the formation of radicals, hence decreasing the degradation efficiency. In addition, more intermediates will exist in the solution upon increasing the initial concentration of MO, and the photons do not easily reach the catalytic sites on the surface of F-Si-TiO_2_/AC at high initial concentrations, resulting in a deceleration of the photodegradation process.

#### 2.6.4. The Effect of Initial pH Conditions

The pH of the solution affects the surface charge of activated carbon, TiO_2_, and dissolved organic molecules. The surface charge of AC and the supported catalyst depends on the zero-charge point (pH_pzc_) and pH of the solution. When pH > pH_pzc_, the surface charges of both TiO_2_ and AC are negative. When pH < pH_pzc_, an opposite trend is observed. According to another research study [47], the zero-charge point of TiO_2_ is 6.2. The pH_pzc_ of AC is between 3 and 7 depending on the preparation method and materials used. In general, when the pH value is lower than the pH_pzc_ of the catalyst, MO at this pH value has an opposite charge to the catalyst, so it is easily adsorbed on the catalyst surface, and the MO degradation efficiency is high, while when the pH is higher than pH_pzc_, the opposite is true, the repulsive charge makes the adsorption of MO on the catalyst surface very low, and the photocatalytic efficiency is significantly reduced.

The effect of the pH conditions on the MO degradation rate by the photocatalysts was studied in the pH range of 2–10. Interestingly, the effect of pH does not exactly meet those expected in terms of the photocatalytic degradation rate toward MO observed for the catalysts. Figure 7d shows as the pH value increases (pH > 2), the photocatalytic degradation rate of MO reaches a maximum at pH 5.5 and then decreases at pH 8–10. The anomalies observed in the degradation efficiency can be attributed to the fact that the pH conditions may change the band gap, which affects the generation and migration of electrons and holes. At optimal pH, it is most conducive to the separation of electron holes. It is also most conducive to the production of active free radicals [48,49]. Therefore, when using F-Si-TiO_2_/AC at pH 5.5, the photocatalytic degradation rate of MO is highest because the radicals generated at this pH can cause the oxidation of MO to the greatest extent. Our subsequent experiments were also performed at pH 5.5.

#### 2.6.5. The Effect of Water Quality Parameters

**Figure 8:**
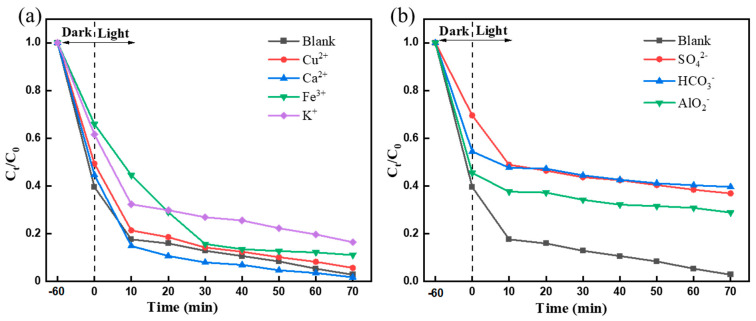
Photocatalytic effect of solutions containing different (**a**) anions and (**b**) cations.

To further evaluate its practical potential, the photocatalytic efficiency of F-Si-TiO_2_/AC under visible light irradiation in solutions containing different anions and cations was studied (Figure 8).

We added CuCl_2_, CaCl_2_, FeCl_3_·6H_2_O, or KCl to the MO solution, and the concentration of these ions was set at 0.01 M. It can be found that all cations have an inhibitory effect on the adsorption of MO by the catalyst. Among all of the studied ions, except for Ca^2+^ (98.32%), which promoted the photocatalytic performance, the other ions slowed down the photocatalytic process (Figure 8). This could be attributed to the active sites of F-Si-TiO_2_/AC being covered with MO molecules and more intermediates, decelerating the generation of electron–hole pairs and inhibiting the formation of radicals.

We added NaHCO_3_, Na_2_SO_4_, or NaAlO_2_ to the MO solution with the concentration of the ion set at 0.01 M, and the results are shown in Figure 8b. The inhibitory effect of these anions (HCO_3_^−^, SO_4_^2−^, or AlO_2_^−^) was in the order of: HCO_3_^−^ > SO_4_^2−^ > AlO_2_^−^. This may be due to an increase in the charge of these anions, which increases the electrostatic repulsion between the ions and reduced the surface adsorption sites of AC and reduced the adsorption rate. Even under the action of other ions, the degradation efficiency of MO remained at 83.6% (K^+^) and 64.0% (HCO_3_^–^) after 70 min. These results show that F-Si-TiO_2_/AC exhibits good potential for practical application in water pollution treatment processes.

#### 2.6.6. Reuse of F-Si-TiO_2_/AC

**Figure 9:**
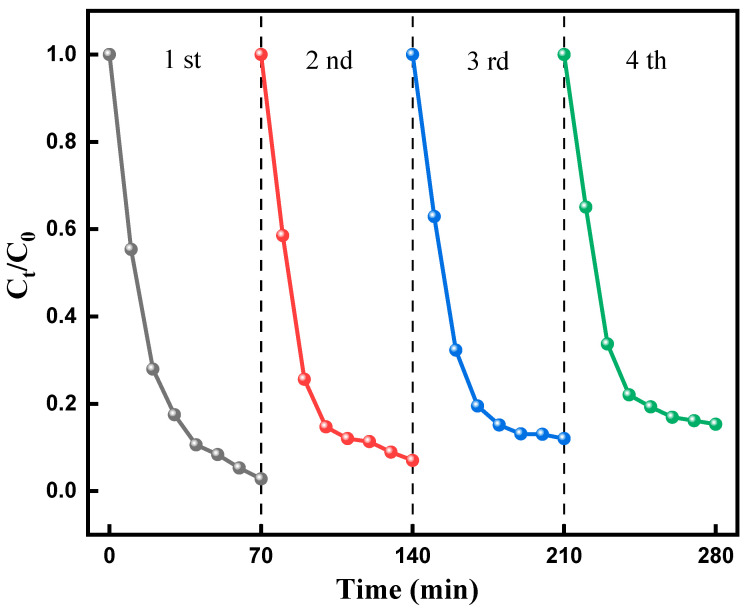
Reusability of F-Si-TiO_2_/AC for the photodegradation of MO.

The stability of the photocatalyst is a vital aspect in terms of evaluating its practical potential, and thus, a series of recycling experiments were carried out using F-Si-TiO_2_/AC for the degradation of MO under irradiation with UV light. After each photodegradation experiment, the F-Si-TiO_2_/AC sample was collected using a centrifuge and calcined at 350 °C for 2 h under a nitrogen atmosphere. Figure 9 shows the degradation efficiency remained at 84.7% after five consecutive runs. Therefore, F-Si-TiO_2_/AC was shown to possess long-term stability and thus exhibit great sustainability for the catalytic degradation of organic pollutants. The slight decrease in the catalytic efficiency may be due to the masking of active sites by the intermediate products of MO and the small loss of F-Si-TiO_2_/AC.

### 2.7. Mineralization of Dyes

**Figure 10:**
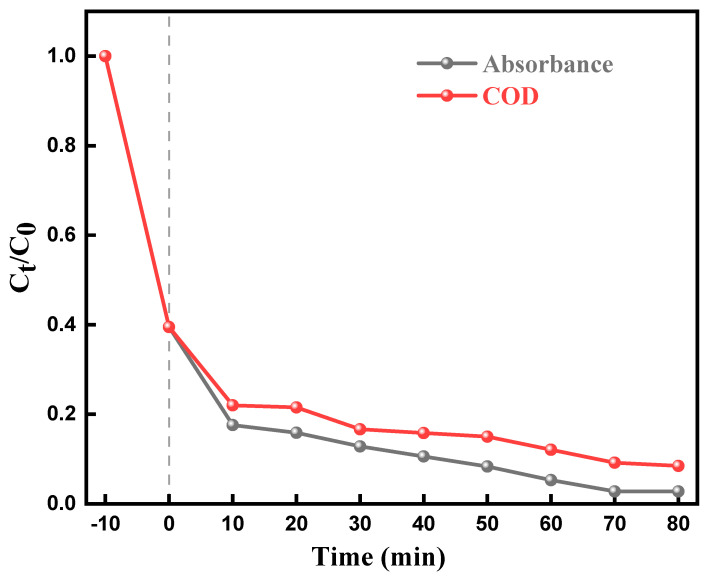
Mineralization percent of MO on F-Si-TiO_2_/AC sample.

The measurement of the fall in the chemical oxygen demand (COD) of irradiated solutions is usually used to monitor the mineralization of dyes. A consistent reduction in COD with the increasement in the time was noted (Figure 10). The percentage mineralization of MO increased with time, reaching a value of 95.14% after 80 min. The percentage mineralization (84.6%) was lower compared to percentage decolorization (99.97%). The presence of smaller, colorless byproducts formed during the decolorization process may contribute to higher COD values. However, these byproducts do not affect the overall decolorization percentage as they do not add color to the system.

### 2.8. Free Radical Capture Experiments of F-Si-TiO_2_/AC

**Figure 11:**
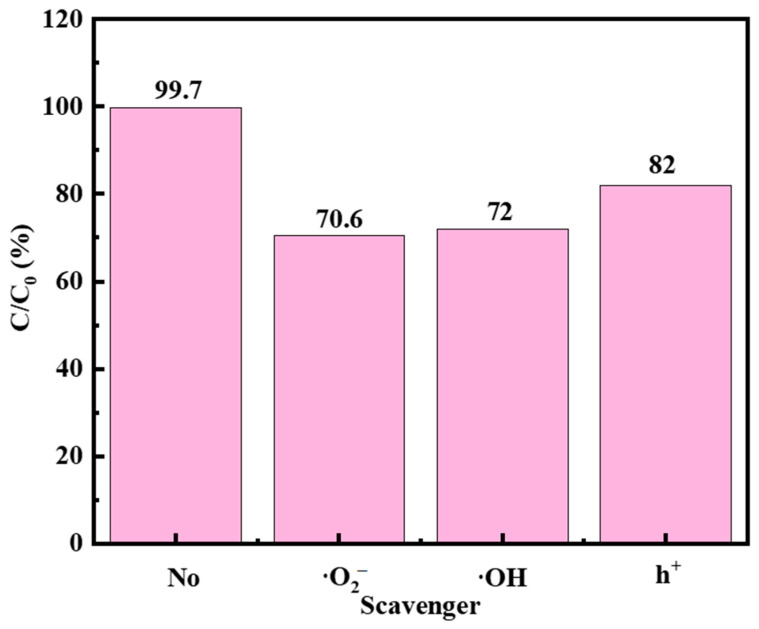
Degradation rate of MO solution by F-Si-TiO_2_/AC in the presence of different scavengers.

An amount of 0.1 mol of the scavenger (EDTA-Na_2_, AIP, and BQ) was added to the solution to capture h^+^, ·OH, and ·O_2_^−^, respectively (Figure 11). Based on the results shown in Figure 11, it was observed that the degradation efficiency of MO only decreased by 17.7% upon addition of h^+^ compared to the control group (without any scavenger) in the photocatalytic solution. However, the introduction of the ·OH scavenger significantly impacted the photocatalytic efficiency, resulting in only 72% decomposition of MO molecules. Similarly, addition of the ·O_2_^−^ scavenger also suppressed the photocatalytic efficiency, resulting in a degradation rate of 70.6% over an hour. The findings indicate that while h^+^ had minimal impact on the photocatalytic degradation effect, the primary active substances in the photocatalytic process were ·O_2_^−^ and ·OH. These observations are significant for understanding the effectiveness of different scavengers for optimizing photocatalysis.

### 2.9. Reaction Intermediates Research of F-Si-TiO_2_/AC

**Figure 12:**
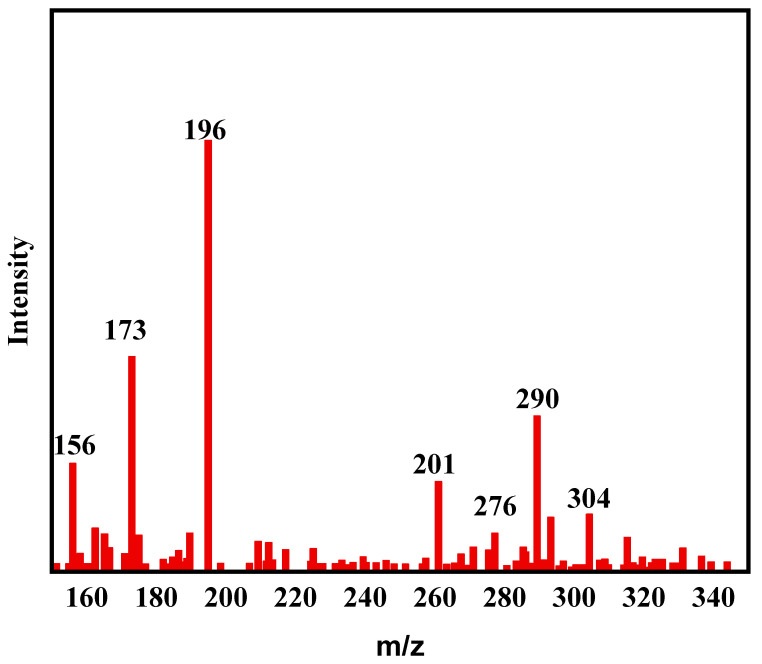
LC–MS spectra for degradation of MO in 60 min.

As a typical azo dye, the degradation process of methyl orange is often accompanied by multiple degradation pathways. To ascertain the degradation pathway of MO, LC-MS was employed in this work. As shown in Figure 12, mass spectrometry reveals seven intermediates derived from MO during degradation.

**Figure 13:**
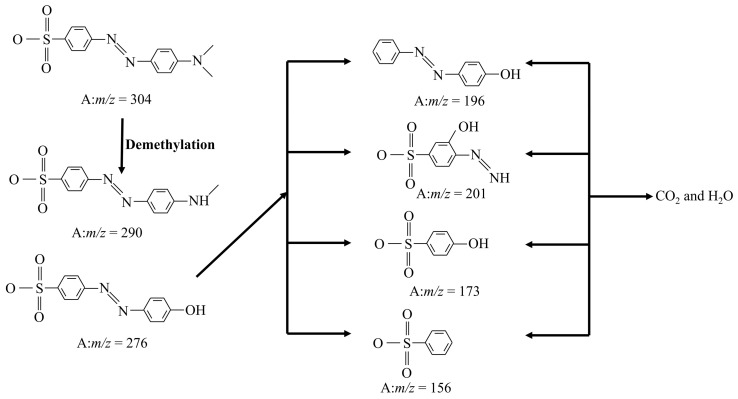
The possible photocatalytic degradation pathways of MO.

Possible degradation pathways for MO are presented in Figure 13. The peak of *m/z* = 304 represents the MO molecule after losing Na. Furthermore, the N-C bond of dimethylaminomine is decomposed, resulting in the formation of new products represented by *m/z* = 290 and 276 [50]. These two intermediates give rise to different products through diverse reaction pathways. A significant portion generates *m/z* = 196, while a smaller fraction indicates that the aromatic ring cleavage transforms into species with *m/z* = 254. The oxidative attack results in the elimination of alkyl groups and the formation of *m/z* = 201 species. The hydroxyl radicals contribute to the creation of *m/z* = 173 species by attacking azo bonds [49]. The intermediate with *m/z* of 157 is a deamination product, and finally, all intermediate products are ultimately converted into CO_2_ and H_2_O [51].

### 2.10. Synergistic Photodegradation Mechanism of F-Si-TiO_2_/AC

**Figure 14:**
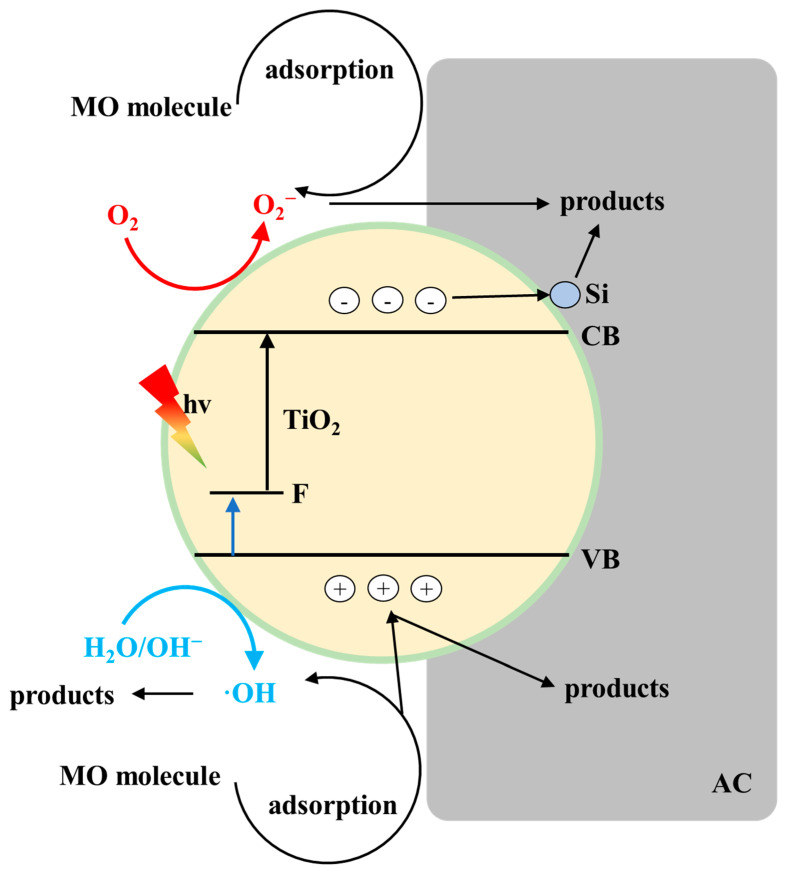
A schematic illustration of the synergistic effect during the photodegradation of MO using F-Si-TiO_2_/AC.

The intriguing phenomenon of enhanced light absorption and photocatalytic efficiency highlights the synergistic effects achieved upon co-doping F and Si. Based on the above results, a possible photocatalytic mechanism is proposed, as shown in Figure 14. First, the MO molecules could be easily adsorbed onto the surface of F-Si-TiO_2_/AC and enhance the molecule concentration near the active sites. Second, the F ions in F-Si-TiO_2_/AC forms a new and lower band gap, which facilitates the occurrence of the photon–hole reaction. When excited upon irradiation with visible light, the photogenerated electrons are transferred more efficiently to the conduction band of TiO_2_, while the holes with high oxidation power are kept in the deep level of the valence band and degrade the MO molecules. Previous studies have shown that Si ions are able capable of capturing a large number of electrons; therefore, the Si doping in F-Si-TiO_2_/AC can act as electron traps. Thus, the photogenerated electrons in the conduction band of TiO_2_ are quickly transferred to AC and inhibit electron/hole recombination by extending the existence time of h^+^. Lastly, AC not only provides the separation ability in F-Si-TiO_2_/AC but also enhances the photocatalytic activity due to the effective separation of h^+^ and e^–^. Therefore, the photocatalytic capability can be greatly improved in F-Si-TiO_2_/AC.

There have been many studies on photocatalytic degradation of MO, and we compare this work with the work of our predecessors, and the results are shown in Table 4.

## 3. Experimental Procedure

### 3.1. Materials and Chemicals

AC, tetrabutyl titanate, ammonium fluoride, sodium hydroxide, anhydrous sodium sulfate, sodium bicarbonate, calcium chloride, anhydrous copper chloride, and potassium chloride were obtained from Macklin Biochemical Technology Co., Ltd. (Shanghai, China). Sodium aluminate calcium chloride was purchased from Aladdin (Shanghai, China). Absolute ethanol, glacial acetic acid, tetraethyl orthosilicate, methyl orange, and ferric chloride hexahydrate were purchased from Sinopharm Chemical Reagent Co., Ltd. (Shanghai, China). All chemicals used in this work were of commercial analytical grade. Deionized (DI) water was used throughout this study.

### 3.2. Preparation of AC

AC was prepared based on a modified procedure described in the literature [59]. AC particles were ground and AC particles with a particle size of <100 nm were screened for use. A certain amount of sieved AC particles was added to DI water, heated at 80 °C for 2 h, and stirred to remove any floating dust. The AC particles were washed repeatedly with DI water until no floating dust was observed and then placed in a blast drying box heated at 100 °C to dry for later use.

### 3.3. Preparation of TiO_2_/AC and F-Si-TiO_2_/AC

**Figure 15:**
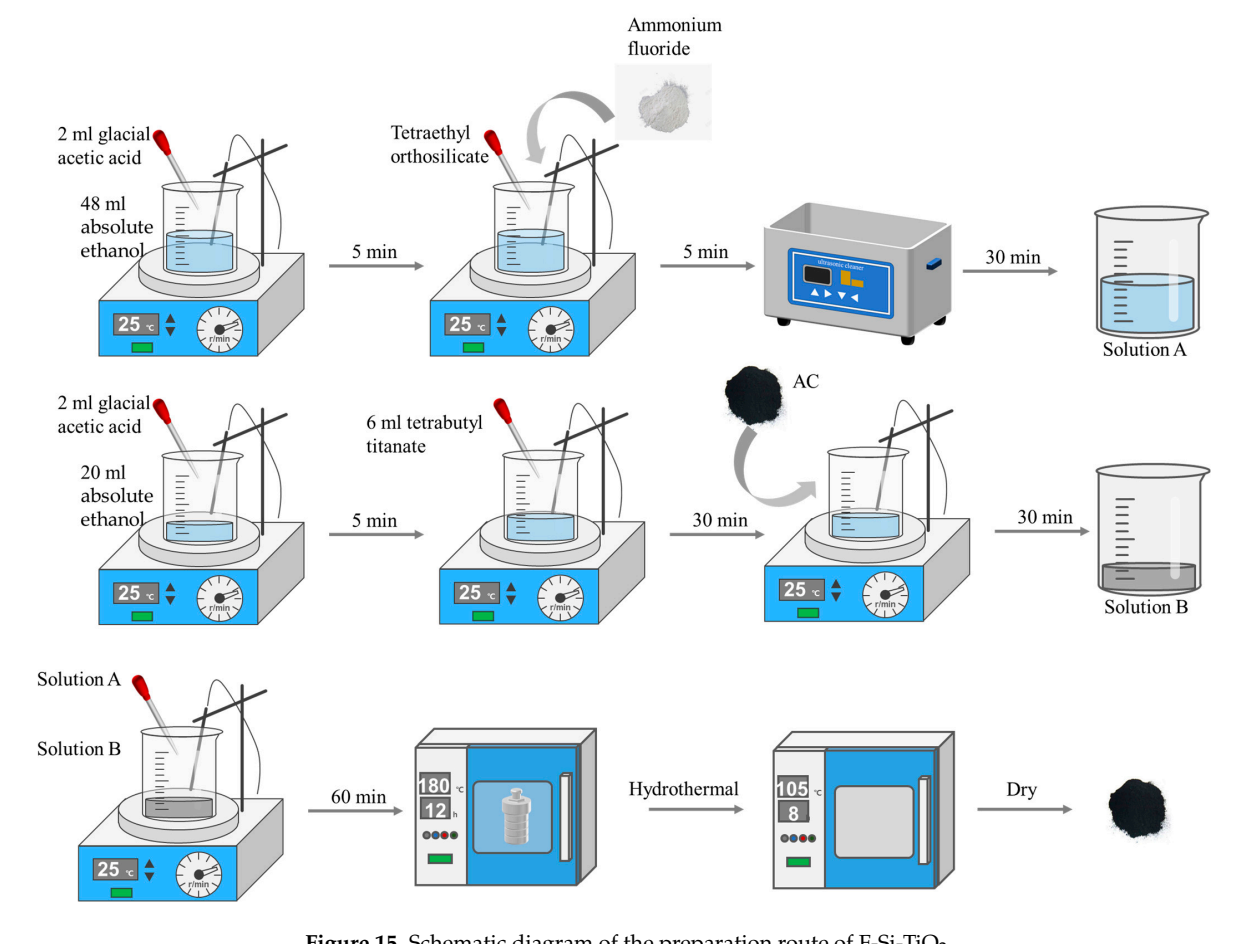
Schematic diagram of the preparation route of F-Si-TiO_2_.

Forty-eight milliliters of absolute ethanol was added to a beaker, and 2.0 mL of glacial acetic acid was added dropwise. Then, ammonium fluoride and tetraethyl orthosilicate were added. The beaker was placed in an ultrasonic cleaner to fully dissolve the ammonium fluoride and tetraethyl orthosilicate to produce solution A. Twenty milliliters of absolute ethanol was poured into another beaker, 2.0 mL of glacial acetic acid was added dropwise, and then 6.8 mL of tetrabutyl titanate was added to the mixed solution of absolute ethanol and glacial acetic acid to produce solution B. One gram of pretreated AC was added to solution B. Solution A was slowly added dropwise to solution B and stirred for 1 h to obtain the precursor solution. The precursor solution was transferred to a Teflon-lined autoclave equipped with a stainless-steel casing for the hydrothermal reaction. The autoclave was naturally cooled to room temperature and centrifuged to collect the pellet, which was then washed with DI water and dried overnight in a convection oven heated at 105 °C for 8 h to obtain F-Si-TiO_2_/AC. TiO_2_/AC was prepared without the addition of ammonium fluoride or tetraethyl orthosilicate in solution A. The detailed preparation route is shown in Figure 15.

### 3.4. Material Characterization

The crystal structures of the prepared samples were identified using X-ray diffraction (XRD) performed on a Bruker’s D8 Advanced X-ray powder diffractometer. Scanning electron microscopy (SEM) images were obtained on a S4800 cold-field emission scanning electron microscope (Hitachi, Tokyo, Japan). The specific surface area, average pore size, and pore volume were determined using Micromeritics’ fully automated specific surface area and microporosity analyzer (ASAP 2020 Plus HD88). An Agilent Cary660 micro-infrared spectrometer was used to analyze the functional groups and chemical bonds in the prepared samples. Ultraviolet–visible spectroscopy (UV–vis) was performed on a Lambda 950 UV–vis spectrophotometer (Perkin Elmer, Waltham, MA, USA).

### 3.5. Adsorption Experiments

F-Si-TiO_2_/AC (10 mg) was dispersed into an aqueous solution of MO (10, 15, 20, and 25 mg/L, 100 mL) and mixed at 500 rpm in the dark. At specified time intervals, 5 mL of the MO solution was removed and filtered, and then using UV–vis spectrophotometer at 463 nm, the adsorption rate of MO was calculated as follows [60]:

Adsorption rate = 100% × (C_0_ − C_t_)/C_0_ (3)

where C_0_ and C_t_ are the initial and remaining concentration of MO, respectively.

### 3.6. Photodegradation Experiments

The photocatalytic activity of the as-prepared catalysts was investigated using the photodegradation of MO utilizing a 300 W high-pressure Xe lamp. Prior to irradiation, F-Si-TiO_2_/AC (10 mg) was dispersed into an aqueous solution of MO (100 mL) and mixed for 60 min in the dark to achieve an adsorption–desorption equilibrium. The mixture was then subjected to visible light irradiation for 70 min. Periodically, a sample of the reaction solution (5 mL) was withdrawn at preset time intervals, and the suspended solids were removed using a centrifuge. The concentration of MO was determined using UV–vis spectroscopy recorded at 463 nm. The degradation efficiency of MO was expressed as follows [61]:

Degradation efficiency = 100% × (C_0_ − C_t_)/C_0_ (4)

where C_0_ and C_t_ are the initial and degraded concentrations of MO, respectively.

## 4. Conclusions

As an attractive photocatalyst, TiO_2_-based materials have been widely applied toward the photocatalytic degradation of organic pollutants. However, traditional TiO_2_-based materials are usually restricted by their unsatisfactory photocatalytic efficiency due to insufficient sunlight absorption, low surface area, and the fast recombination of photo-induced electron–hole pairs. F-Si-TiO_2_/AC can lead to the formation of new energy levels in the band gap, extend the spectral response properties, improve the surface area, increase the adsorption ability, is easily separated, and reduces the recombination rate of electron-hole pairs. Therefore, the photocatalytic performance of F-Si-TiO_2_/AC was remarkably increased for MO. The corresponding removal rate of MO reached 97.7%. The process can be hindered by impurity ions, such as Ca^2+^, Fe^3+^, Cu^2+^, K^+^, SO_4_^2−^, HCO_3_^−^, and AlO_2_^−^. The pathway of the degradation process has been illustrated and ·OH plays a major role in cleaving the MO molecules. After five reuse cycles, the removal rate of MO slightly dropped to 84.7%. Its good reusability and cost-effective properties have proven it to be highly applicable for wastewater treatment.

## Figures and Tables

**Table 1:** Physical properties of as-prepared P25, TiO_2_/AC, F-TiO_2_/AC, Si-TiO_2_/AC, and F-Si-TiO_2_/AC.

Samples	Crystalline Size (nm)	A and B	C	2Θ (101)	Predominant Facet (%)	FWHM (101)	Interplanar Distance
TiO_2_/AC	10.2	3.818	9.467	25.201	58.8	1.487	3.536
F-TiO_2_/AC	9.4	3.812	9.459	25.098	63.5	1.502	3.550
Si-TiO_2_/AC	8.2	3.802	9.482	25.158	63.3	1.500	3.541
F-Si-TiO_2_/AC	9.4	3.824	9.502	25.102	69.6	1.516	3.549

**Table 2:** The corresponding surface area (S_BET_), pore volume, and average pore size of P25, TiO_2_/AC, and F-Si-TiO_2_/AC.

Samples	S_BET_ (m^2^·g^−1^)	Pore Volume (cm^3^·g^−1^)	Average Pore Size (nm)
AC	672.623	0.486	2.846
P25	62.639	0.341	2.177
TiO_2_/AC	618.518	0.428	2.765
F-Si-TiO_2_/AC	591.285	0.398	2.690

**Table 3:** Photocatalytic degradation observed using the different samples.

Sample	K(×10^–4^/min) for MO Visible Light Irr.	Band Gap and Mid-Gap Level Energy (eV)	F: TiO_2_	Si: TiO_2_	Degradation (%)
TiO_2_	12.9	3.20	0	0	14.3
TiO_2_/AC	57.8	2.85	0	0	67.0
Si-TiO_2_/AC	30.5	3.32	0	0.1	58.2
F-TiO_2_/AC	191.4	2.80	0.6	0	89.8
F-Si-TiO_2_/AC	318.0	2.70	0.6	0.1	97.2

**Table 4:** Degradation times and efficiencies resulting from the catalytic photodegradation of RhB dye using some of the previously reported photocatalysts and F-Si-TiO_2_/AC.

Photocatalysts	Catalyst Dosage (mg/100 mL)	Initial Dye Concentration (mg/L)	Rection Time (min)	Efficiency (%)	Ref
Pd/TiO_2_	100	20	60	100	[52]
TiO_2_	100	20	75	72.79	[53]
C/TiO_2_	50	40	200	99.7	[54]
Cr-TiO_2_	50	1.6	300	78	[55]
P-C-TiO_2_	100	10	14	95	[56]
Ag@AgP_3_O_4_	100	10	30	80	[57]
ZnO	30	10	30	94.5	[58]
F-Si-TiO_2_/AC	10	10	70	99.7	This work

## Data Availability

Data available on request due to restrictions, e.g., privacy or ethical.

## Sample Availability

Samples of the compounds are available from the authors.
